# Divergent dFC stability of DMN and SMN in narcolepsy

**DOI:** 10.3389/fnins.2026.1746322

**Published:** 2026-06-08

**Authors:** Wenyi Li, Lijing Jia, Qiuxing Hong, Xingyu Wang, Zhenzhong Li, Zuojun Geng

**Affiliations:** 1 Department of Neurology, The Second Hospital of Hebei Medical University, Shijiazhuang, Hebei, China; 2 The Key Laboratory of Neurology, Hebei Medical University, Ministry of Education, Shijiazhuang, Hebei, China; 3 Department of Radiology, The Affiliated Hospital of Southwest Medical University, Luzhou, Sichuan, China; 4 Department of Radiology, The Second Hospital of Hebei Medical University, Shijiazhuang, Hebei, China

**Keywords:** default mode network, dynamic functional connectivity, narcolepsy, sleep efficiency, somatomotor network, state instability

## Abstract

Narcolepsy type 1 (NT1) is characterized by profound sleep-wake state instability, pointing to a fundamental dysregulation of large-scale brain network dynamics. To elucidate this, we assessed whole-brain dynamic functional connectivity (dFC) stability using resting-state fMRI in 27 patients with NT1 and 25 matched healthy controls. Our analysis revealed a pattern of opposing alterations: patients exhibited significantly increased dFC stability within the bilateral somatomotor network (SMN), concurrent with decreased stability in the medial prefrontal default mode network (Default_PFCm). These opposing alterations were clinically relevant, as increased SMN stability correlated with poorer objective sleep efficiency, and decreased Default_PFCm stability was similarly associated with lower objective sleep efficiency. Here, we identify for the first time this coexisting neural signature of SMN hyper-stability and Default_PFCm instability in NT1. By simultaneously destabilizing higher-order cognitive networks and disinhibiting primary sensorimotor processing, orexin deficiency may contribute to a synergistic dysregulation that blurs sleep-wake boundaries. The divergence in dynamic network stability provides a novel systems-level framework for understanding state instability in NT1.

## Introduction

Narcolepsy, a central disorder of hypersomnolence resulting from the loss of hypocretin/orexin neurons, is characterized by a fundamental dysregulation of sleep-wake state boundaries. Beyond the core symptoms of excessive daytime sleepiness and cataplexy, patients commonly experience severe nocturnal sleep fragmentation, pointing to a core deficit in the central mechanisms governing the stability of sleep, and wakefulness (Bassetti et al., 2019). Resting-state functional magnetic resonance imaging (rs-fMRI) has offered valuable insights into its neural underpinnings, with numerous studies suggesting aberrant integration within large-scale brain networks, including the default mode and salience networks (Fulong et al., 2020; Xu et al., 2023). However, much of this work relies on the assumption of stationarity in functional connectivity over the scanning period, potentially overlooking the time-varying properties of brain function that may be more directly relevant to the state instability central to the disorder (Wada et al., 2019).

Recent studies have begun to investigate dynamic functional connectivity (dFC) in narcolepsy and related hypersomnolence disorders (Mengmeng et al., 2025, 2026), reporting altered temporal properties of large-scale brain networks, including abnormal occurrence and persistence of connectivity states. These findings suggest impaired flexibility of network dynamics and disrupted sleep–wake regulation, particularly involving interactions among default mode, somatomotor, and visual systems. However, these approaches primarily characterize discrete connectivity states and their temporal properties, providing limited insight into the stability of connectivity patterns at a continuous temporal scale.

Dynamic functional connectivity (dFC) analysis provides a framework for characterizing the time-varying properties of brain function by assessing temporal fluctuations in functional coupling between regions. Among these approaches, dFC stability offers a complementary perspective by quantifying the temporal consistency of connectivity patterns across time windows, thereby capturing the balance between persistence and flexibility of brain network organization. Metrics such as dFC stability, which reflects the brain's capacity to maintain a functional state or switch flexibly between states, offer a compelling theoretical approach for investigating conditions with state control deficits, such as narcolepsy (Li et al., 2020; Sun et al., 2022). Under normal resting-state conditions, higher-order association networks such as the default mode network typically exhibit relatively higher temporal stability, whereas primary sensory and motor regions show greater variability, reflecting differences in intrinsic processing timescales and functional roles. Despite its potential, a systematic examination of whole-brain dFC stability in narcolepsy remains limited, and its alteration profile is poorly understood.

Our investigation into whole-brain dynamic functional connectivity (dFC) in narcolepsy revealed a key finding: an opposing alteration in temporal stability between two major networks. Compared to 25 matched healthy controls, 27 patients exhibited decreased stability in the bilateral prefrontal default mode network (Default_PFCm) and concurrently increased stability in the bilateral somatomotor network (SMN). This divergent pattern suggests a disruption of the canonical hierarchical organization of brain network stability, in which higher-order associative networks typically exhibit greater temporal stability, whereas primary sensory and motor systems remain relatively more variable.

We propose that these changes reflect complementary aspects of the disorder. On one hand, Default_PFCm instability may contribute to impaired arousal and attention. On the other hand, SMN hyper-stability likely indicates reduced temporal flexibility within sensorimotor systems, which may increase susceptibility to sleep fragmentation. This interpretation is strengthened by our subsequent findings that increased stability in the bilateral SMN was significantly correlated with poorer objective sleep efficiency, while decreased stability in the right prefrontal Default_PFCm was also associated with impaired sleep quality.

Thus, by focusing on brain dynamics, our study uncovers a novel neural signature of narcolepsy—a divergence in functional stability across networks—that offers a new framework for understanding its core symptom of state instability. By extending prior state-based dFC findings toward a stability-based characterization, this study provides a complementary perspective on the temporal organization of brain networks in NT1. By elucidating the dynamic functional architecture underlying the core symptoms of NT1, this study aims to provide insights into the neural mechanisms of conscious state regulation, a key question in cognitive neuroscience and biological psychiatry.

## Materials and methods

### Participants

The study included 30 individuals with narcolepsy type 1 and 25 healthy controls matched for age and sex. Due to excessive head motion during fMRI scanning (translation or rotation exceeding 2.0 mm), three patients were excluded from further analysis, resulting in a final cohort of 27 patients and 25 controls.

All narcolepsy patients were diagnosed according to the International Classification of Sleep Disorders, Third Edition (ICSD-3). The diagnostic criteria comprised: (1) excessive daytime sleepiness lasting at least 3 months, accompanied by either clear cataplexy (as assessed by two neurologists) or a cerebrospinal fluid hypocretin-1 level ≤ 110 pg/mL; and (2) a mean sleep latency of ≤ 8 min with two or more sleep-onset REM periods (SOREMPs) on the Multiple Sleep Latency Test (MSLT), or one SOREMP on MSLT together with a nocturnal SOREMP observed during prior polysomnography (PSG).

Healthy controls were screened to exclude individuals with a history of narcolepsy symptoms, neurological conditions, or psychiatric disorders. Participants had not taken any medications affecting sleep-wake function for at least 4 weeks before the study. In addition, patients with clinically significant obstructive sleep apnea–hypopnea syndrome (OSAHS) were excluded to minimize potential confounding effects of comorbid sleep-disordered breathing. Structural MRI results confirmed the absence of brain lesions in all subjects. The study was approved by the Institutional Ethics Committee (Approval No.: 2023-R437), and written informed consent was provided by every participant.

### Neuroimaging acquisition and processing

High-resolution T1-weighted and resting-state functional MRI data were collected on a 3T GE Signa scanner (General Electric Healthcare, USA) equipped with a 64-channel head coil. Structural imaging used a 3D T1-weighted fast spoiled gradient-recalled echo (FSPGR) sequence with the following parameters: repetition time (TR) = 9 ms, echo time (TE) = 3.4 ms, flip angle = 15, inversion time (TI) = 450 ms, slice thickness = 1 mm, and field of view (FOV) = 224 × 224 mm^2^. Functional images were acquired using a gradient-echo echo-planar imaging (GRE-EPI) sequence sensitive to blood oxygen level-dependent (BOLD) contrast (TR = 2,000 ms, TE = 30 ms, flip angle = 60, slice thickness = 3.5 mm, FOV = 224 × 224 mm^2^). The entire resting-state scan lasted 8 min, yielding 240 volumes. Participants exhibiting head motion exceeding 2.0 mm translation or 2.0°rotation were excluded.

### Data preprocessing and dynamic functional analysis

All MRI data were processed using the DPABISurf pipeline (Yan et al., 2021), which integrates fMRIPrep (v23.1.4) for standardized preprocessing. For anatomical data, T1-weighted images underwent bias correction, skull stripping, tissue segmentation, and cortical surface reconstruction (FreeSurfer v7.3.2), followed by nonlinear normalization to the MNI152NLin2009cAsym template.

For functional data, the first 10 volumes were discarded, followed by slice timing and head motion correction. Six rigid-body motion parameters were estimated, and time points with framewise displacement (FD) > 0.5 mm were identified as motion outliers. Functional images were co-registered to T1-weighted images and projected onto cortical surfaces in fsaverage5 space.

Subsequent preprocessing within the DPABISurf pipeline included nuisance regression [Friston-24 motion parameters, WM and CSF signals, linear detrending, temporal band-pass filtering (0.01–0.1 Hz), and surface-based spatial smoothing (FWHM = 6 mm)]. All analyses were conducted in surface space.

Dynamic functional connectivity (dFC) stability was estimated using the Temporal Dynamic Analysis module in DPABISurf based on the FunSurfWCF framework. A sliding-window approach was applied (window size = 64 s, step size = 4 s), and linear detrending was performed within each window. Functional stability at each vertex was quantified as Kendall's concordance coefficient across these dFC maps. This process was repeated across all vertices to obtain a whole-cortex dynamic functional stability map for each subject. Individual stability maps were standardized to z-scores within the cortex mask and spatially smoothed with a 6-mm FWHM Gaussian kernel in fsaverage5 space.

### Reproducibility analyses

To evaluate the robustness of our findings to sliding-window parameter choices, we repeated the between-group comparisons with varying window sizes (30 s, 60 s, 80 s) and step sizes (2 s, 4 s). All statistical maps were thresholded identically (cluster-level *p* < 0.05, Monte Carlo corrected) and overlaid on the MNI152 template for visualization. Anatomical labels were consistently assigned using the Yeo2011 atlas.

### Clinical assessments

All participants underwent clinical evaluation within 1 week prior to MRI. Demographic data, including age, sex, height, and weight were recorded. For patients with NT1, disease duration and relevant clinical features were documented. Daytime sleepiness was assessed using the Epworth Sleepiness Scale (ESS). Depressive and anxiety symptoms were evaluated using the HAMD and HAMA scales. All patients with NT1 underwent overnight polysomnography (PSG) and multiple sleep latency testing (MSLT). Total sleep time (TST) and time in bed (TIB) were recorded, and sleep efficiency (SE = TST / TIB × 100 %) was calculated. Mean sleep latency and sleep-onset REM periods (SOREMPs) were obtained from MSLT. Sleep efficiency was selected as the primary clinical correlation variable. Additional exploratory correlations were performed with ESS, MSLT-related measures, HAMD, and HAMA scores.

### Supplementary seed-based dynamic functional connectivity analysis

To further investigate the dynamic interactions associated with functional stability abnormalities, we performed a supplementary seed-based dynamic functional connectivity (dFC) analysis. Seed regions were defined based on clusters showing both significant stability differences and associations with sleep efficiency, including LH2109, RH6449, and RH6681. Dynamic connectivity between each seed and all cortical vertices was computed using a sliding-window approach, and its temporal variability was quantified as the standard deviation of Fisher z-transformed connectivity values (stdzFC), consistent with the main analysis.

### Statistical analysis

Demographic variables were reported as mean ± standard deviation or counts and analyzed using independent-sample t-tests or χ^2^ tests in SPSS Statistics (v26.0).

For neuroimaging analyses, vertex-wise between-group comparisons of dFC stability were performed using a two-sample t-test within the DPABI toolbox, with age and gender included as covariates of no interest. Statistical significance was determined using a cluster-level inference approach based on Monte Carlo simulation (10,000 iterations), with an initial vertex-level threshold of *p* < 0.001 and a cluster-level threshold of *p* < 0.05 (corrected for multiple comparisons across the whole brain) based on the estimated intrinsic smoothness of the data.

Significant clusters were anatomically labeled by projecting the Yeo2011 17-network cortical parcellation onto the fsaverage5 surface. This labeling procedure was performed *post hoc* and served only for anatomical localization and network attribution.

Pearson correlation analyses were conducted to examine the relationships between dFC stability indices and sleep efficiency, with results corrected for multiple comparisons using the false discovery rate (FDR; *q* < 0.05).

For the supplementary seed-based dFC analysis, between-group comparisons were performed using a vertex-wise general linear model controlling for age and sex, and statistical significance was determined using the same cluster-level inference approach as described above.

## Results

### Demographic and clinical characteristics

The study included 27 patients with narcolepsy (17 males/10 females; mean age = 24.96 ± 9.0 years) and 25 healthy controls (15 males/10 females; mean age = 23.96 ± 6.5 years). There were no significant differences between groups in age (independent samples t-test, *t* = 0.05, *p* = 0.96) or sex distribution (χ^2^ test, χ^2^ = 0.05, *p* = 0.83), indicating successful matching on these demographic variables. Further details regarding demographic and clinical characteristics can be found in Supplementary Table 1.

### Between-group differences in dFC stability

**Figure 1:**
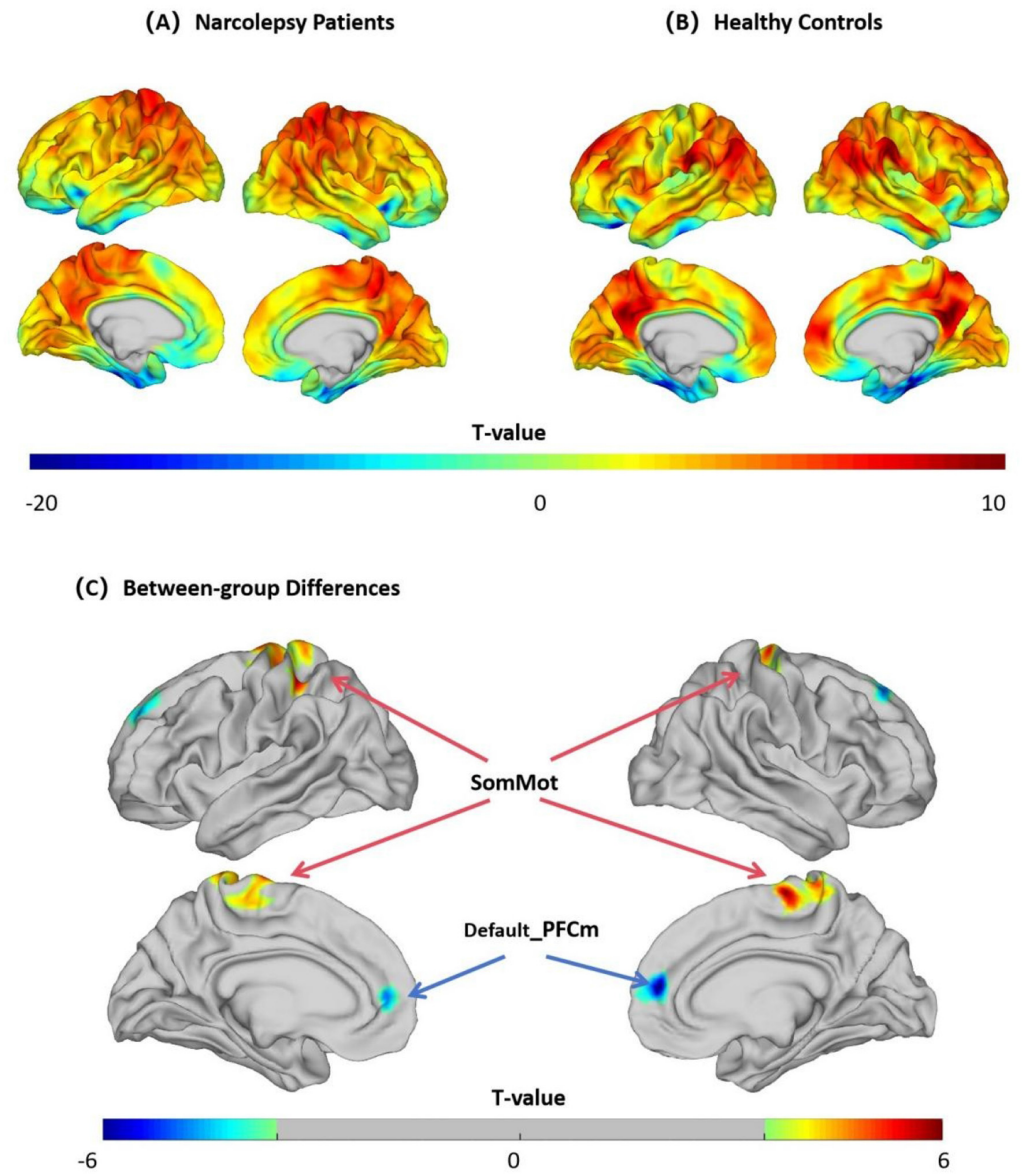
*Between-group differences in intrinsic brain activity stability between narcolepsy patients and healthy controls*. **(A)** Spatial distribution of intrinsic functional stability in narcolepsy patients from one-sample *t*-test results. **(B)** Comparable stability patterns in healthy controls. Color bar for **(A)** and **(B)** represents *T*-values ranging from −20 to 10, with blue indicating lower stability and red indicating higher stability. **(C)** Between-group differences showing significantly increased stability in somatomotor network (SomMot) and decreased stability in prefrontal default mode network (Default_PFCm) in patients compared to controls (two-sample *t*-test, Monte Carlo corrected, *p* < 0.05). Color bar for **(C)** represents *T*-values of group differences ranging from −6 to 6, with blue indicating lower stability and yellow/red indicating higher stability in patients relative to controls.

This analysis revealed significant alterations in dFC stability in patients with narcolepsy compared to healthy controls. Patients exhibited increased dFC stability within extensive clusters of the bilateral somatomotor networks (SomMot). Conversely, significantly decreased dFC stability was observed within several clusters of the prefrontal components of the default mode network (as illustrated in Figure 1). The location, size, and peak statistics of these significant clusters are detailed in Supplementary Table 2. These findings suggest a divergent pattern of temporal brain dynamics in narcolepsy, characterized by hyper-stability in sensorimotor processing regions and hypo-stability in higher-order cognitive prefrontal areas.

### Correlation between dFC stability and sleep efficiency

**Figure 2:**
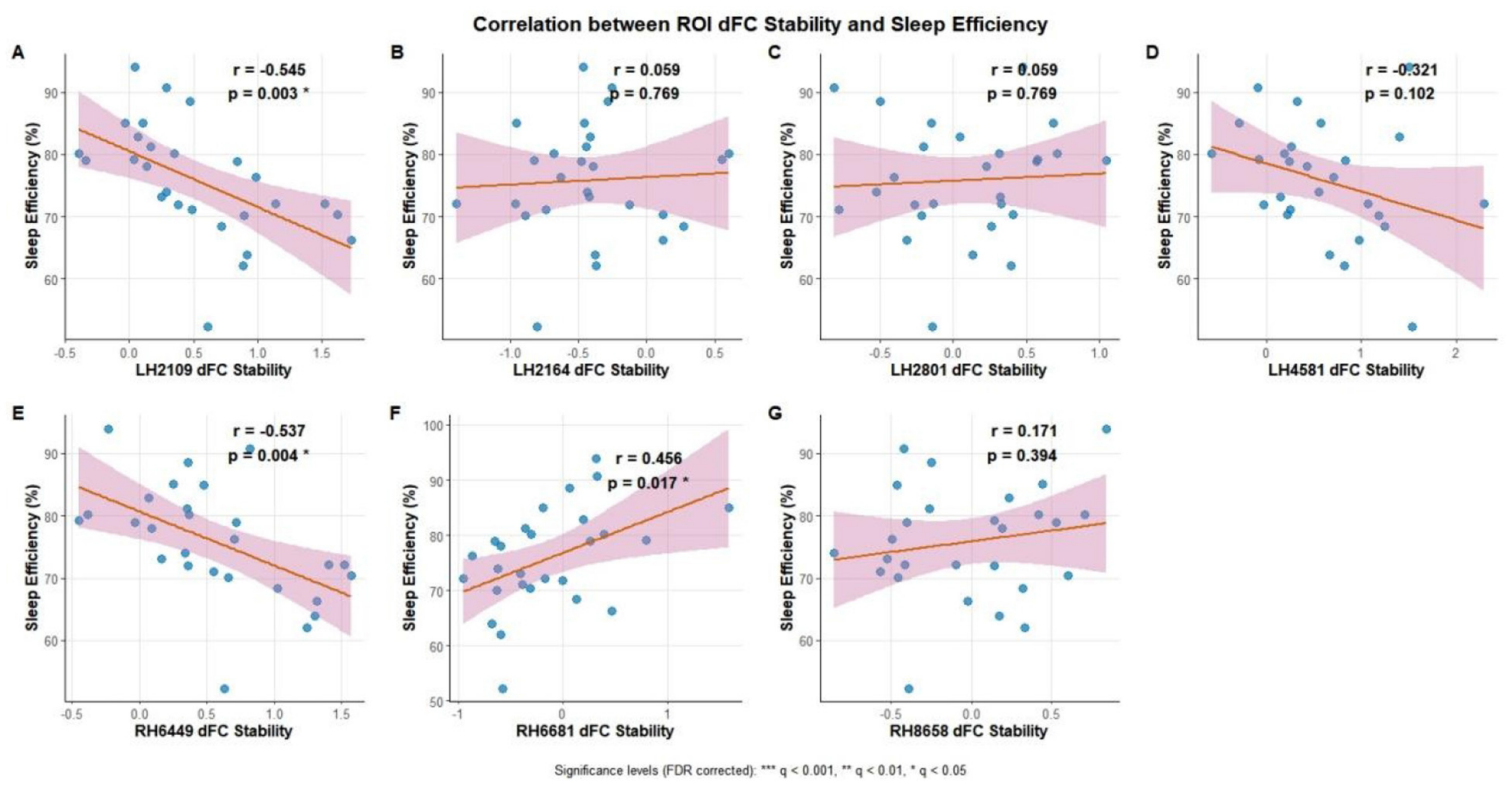
*Scatter plots showing the relationship between dFC stability in group-different ROIs and sleep efficiency*. Blue circles represent individual data points, orange lines show linear regression fits with 95% confidence intervals (shaded). Significance levels after FDR correction: *q < 0.05. Negative correlations were observed in somatomotor regions **(A, E)**, while a positive correlation was found in the prefrontal default mode region **(F)**. No significant associations were detected in the remaining regions **(B, C, D, G)**.

We further investigated the clinical relevance of these dFC stability alterations by correlating the stability indices with polysomnography-derived sleep efficiency. Following FDR correction, a significant negative correlation was retained between sleep efficiency and dFC stability in the left somatomotor cluster (Peak Index: 2,109; *r* = −0.545, *p*_FDR_ < 0.05) and the right somatomotor cluster (Peak Index: 6,449; *r* = −0.537, *p*_FDR_ < 0.05). This indicates that higher dynamic stability in the somatomotor cortex was associated with poorer sleep quality. Conversely, a significant positive correlation was found between sleep efficiency and stability in a right prefrontal default mode cluster (Peak Index: 6,681; *r* = 0.456, *p*_FDR_ < 0.05), suggesting that greater instability (lower dFC stability) in this region is also linked to impaired sleep. Correlations for other regions did not survive FDR correction (as illustrated in Table 1 and Figure 2).

**Table 1:** Correlations between dFC stability in group-different ROIs and sleep efficiency.

ROI	Brain region	Peak index	*r*	*p*-value	*q*-value (FDR)	Significance
LH2109	Left somatomotor cortex	2,109	−0.545	0.0033	0.0116	^**^
LH2164	Left default PFC	2,164	0.059	0.7685	0.8050	
LH2801	Left default PFC	2,801	0.059	0.7693	0.8050	
LH4581	Left somatomotor/DorsAttn posterior	4,581	−0.321	0.1021	0.1790	
RH6449	Right somatomotor cortex	6,449	−0.537	0.0039	0.0116	^**^
RH6681	Right medial prefrontal cortex	6,681	0.456	0.0167	0.0292	^*^
RH6858	Right default PFC	6,858	0.171	0.3944	0.5510	

ROIs were identified as showing significant between-group differences (narcolepsy vs. controls) in dFC stability. dFC = dynamic functional connectivity; FDR = False Discovery Rate correction for multiple comparisons. Significance levels are based on uncorrected *p*-values: ^**^p < 0.01, ^*^p < 0.05.

Additional exploratory analyses were performed with other clinical measures, including ESS, MSLT-related indices, HAMD, and HAMA scores. Although several trend-level associations were observed, none survived correction for multiple comparisons. Detailed results are provided in Supplementary Table 4.

### Reproducibility of dFC stability findings

**Figure 3:**
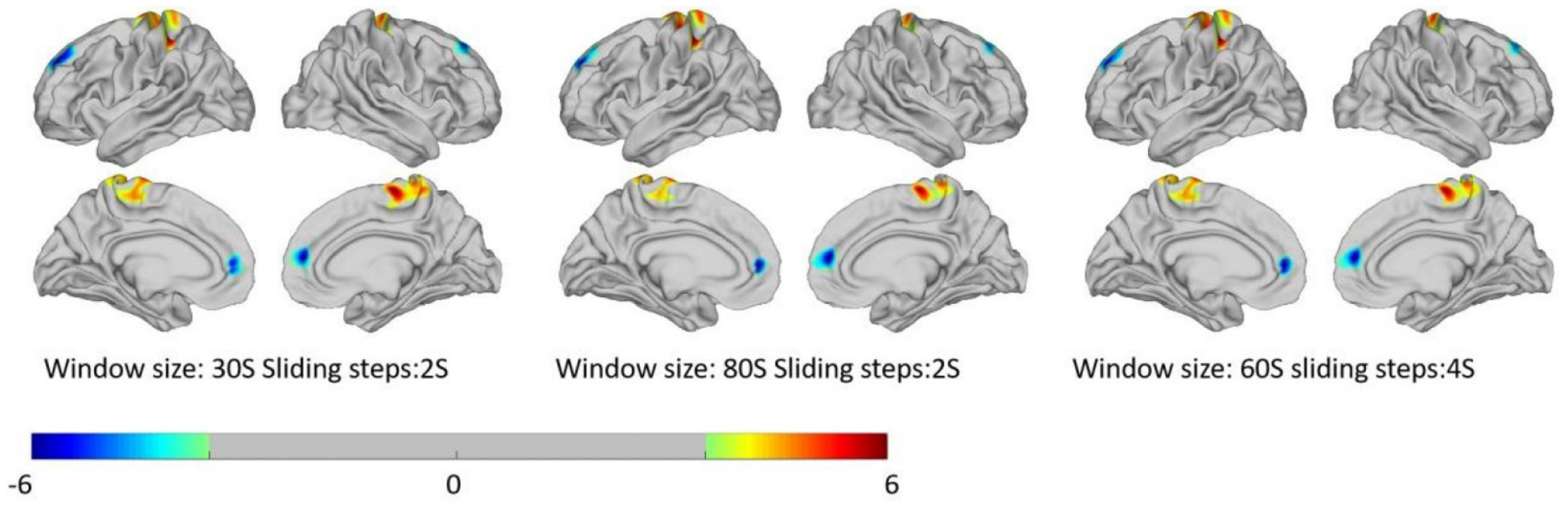
*Reproducibility analyses of dynamic functional stability with different sliding-window parameters*. Between-group comparisons were performed using varying window sizes (30 s, 60 s, and 80 s) and sliding steps (2 s and 4 s) to examine the robustness of functional stability analyses. The consistent spatial patterns across different parameter combinations demonstrate the reproducibility of our primary findings, particularly showing: (1) increased stability in somatomotor networks (warm colors) and (2) decreased stability in prefrontal default mode regions (cool colors) in narcolepsy patients compared to healthy controls. All results are thresholded at *p* < 0.05 (Monte Carlo corrected). Color bars represent *T*-values ranging from −6 to 6.

To assess the robustness of our primary findings, we conducted a series of supplementary analyses using varying sliding-window parameters (window sizes: 30 s, 60 s, 80 s; step sizes: 2 s, 4 s). The spatial pattern of between-group differences remained highly consistent across all parameter combinations. Specifically, the increased dFC stability in the somatomotor networks and the decreased stability in the prefrontal default mode regions were reliably reproduced (all results thresholded at *p* < 0.05, Monte Carlo corrected). This demonstrates that the observed alterations in dFC stability are not dependent on the specific choice of windowing parameters and confirms the robustness of our results (as illustrated in Figure 3).

### Supplementary seed-based dynamic functional connectivity results

Supplementary dFC analysis revealed that both left and right somatomotor seeds (LH2109 and RH6449) showed altered dynamic coupling primarily with visual regions, demonstrating a consistent pattern across hemispheres. In addition, the right medial prefrontal seed (RH6681) exhibited altered dynamic connectivity with a dorsal attention region. Regions with abnormal functional stability were associated with altered dynamic functional connectivity with specific large-scale brain networks (Supplementary Table 3, cluster-wise results).

## Discussion

Our exploration of dynamic functional connectivity (dFC) stability in Narcolepsy Type 1 (NT1) points to a novel, distinctive phenomenon—a divergence in stability across major brain networks. This divergent pattern provides a concise systems-level model for understanding the core phenotype of state instability in NT1. We found a clear double dissociation: patients showed abnormally increased dFC stability in the somatomotor network (SMN) but significantly decreased stability in the medial prefrontal default mode network (Default_PFCm). Critically, the extent of these opposing changes correlated with objective sleep efficiency, underscoring their clinical relevance and pointing to complementary pathophysiological pathways. Sleep efficiency was of particular relevance because it provides an objective index of nocturnal sleep continuity and fragmentation, two core manifestations of sleep–wake instability in NT1. Notably, functional stability does not necessarily reflect increased functional strength, but rather the temporal consistency of dynamic functional connectivity patterns across time windows.

The most consistent finding of this study is the abnormal hyper-stability observed within the somatomotor network (SMN). Under normal conditions, the SMN undergoes dynamic reconfiguration in response to internal and external stimuli. Under typical resting-state conditions, higher-order association networks—particularly the default mode network (DMN)—tend to exhibit relatively higher functional stability, whereas primary sensory and motor regions show greater temporal variability. This distribution is thought to reflect differences in intrinsic processing timescales and functional roles. In NT1, however, this increased stability suggests a relative reduction in temporal flexibility, which may hinder appropriate state transitions across wakefulness and sleep. This reduced flexibility may contribute to persistent sensorimotor engagement and increased sensitivity to internal or external perturbations. Such alterations may reflect impaired regulation of sensorimotor processing across sleep–wake transitions, rather than a direct manifestation of specific perceptual symptoms. Importantly, stability within bilateral SMN clusters (Peak Index: LH2109, RH6449) demonstrated a significant negative correlation with sleep efficiency, indicating that greater SMN rigidity was associated with worse sleep efficiency. A plausible mechanism is that the inability of the SMN to effectively deactivate or disengage during wakefulness persists into sleep, leading to fragmented sleep microstructure and impaired sleep maintenance.

Supplementary seed-based dynamic functional connectivity analysis further demonstrated that both left and right SMN seeds exhibited altered dynamic coupling predominantly with visual regions, showing a spatially constrained but consistent pattern across hemispheres. This finding suggests that SMN hyper-stability is embedded within altered dynamic coupling between somatomotor and visual systems, rather than representing an isolated local effect. These findings are further contextualized by a growing body of neuroimaging evidence linking SMN dysfunction to sleep disturbances across various conditions. Ma et al. (2024) reported enhanced cerebral blood flow (CBF) similarities within the SMN and between the SMN and higher-order associative networks in chronic insomnia, which were correlated with clinical symptoms and cognitive deficits. This convergence of evidence is strengthened by studies focusing on functional connectivity (FC). For instance, increased FC within the SMN has been positively correlated with poorer subjective sleep quality in healthy young males, with specific associations found between PSQI scores and connectivity of the paracentral lobule, precentral gyrus, supplementary motor area, and postcentral gyrus (Bai et al., 2022). Furthermore, increased FC between the SMN and the cingulo-opercular control network has been shown to partially mediate the relationship between psychological stress and poor sleep quality (Zhang et al., 2020). Collectively, these findings from static and perfusion-based measures provide converging evidence for SMN involvement, while the present study extends this framework by specifically highlighting abnormalities in temporal stability and time-varying functional coupling. Polysomnography studies have reported fragmented sleep across stages in NT1, reflecting global sleep–wake instability, which is consistent with the network-level abnormalities observed in the present study (Xu et al., 2017). This pattern dovetails with SMN hyper-stability, which may increase vulnerability to micro-arousals and unstable sleep–wake transitions.

Conversely, our findings indicate reduced dynamic stability—reflecting increased temporal variability—in the medial prefrontal subdivision of the default mode network (Default_PFCm), suggesting a diminished capacity to sustain stable cognitive states. As a central hub for self-referential processing and cognitive control, dysfunction in this region may compromise the maintenance of internally directed cognitive processes (Mak et al., 2017). This result aligns with previously documented impairments in sustained attention and executive function in NT1 (Cano et al., 2024; Kim et al., 2008; van Holst et al., 2018). Previous neuroimaging studies have also reported altered default mode network connectivity in NT1, particularly involving medial prefrontal regions associated with attention and cognitive control. Correlation analyses with formal cognitive assessments were not performed, primarily due to limited variability in screening scores (e.g., MMSE, MoCA) and the subjective nature of these measures. Previous studies (Vu et al., 2011; Zamarian et al., 2014) have also reported that subjective cognitive complaints in NT1—particularly regarding attention—tend to correlate more strongly with transient sleepiness and affective symptoms than with performance-based cognitive measures. Given these considerations, we focused on relationships with physiologically grounded measures. We observed a significant positive correlation between Default_PFCm instability and reduced sleep efficiency, suggesting a close relationship between prefrontal dysregulation and sleep quality. This interpretation is further supported by supplementary findings showing altered dynamic coupling between the Default_PFCm and dorsal attention regions.

Notably, supplementary analysis revealed that the right medial prefrontal seed (RH6681) exhibited altered dynamic coupling with a dorsal attention region. This pattern suggests disrupted temporal coordination between internally oriented (default mode) and externally oriented (attention) systems, providing a network-level context for Default_PFCm instability. We propose that instability in the Default_PFCm may disrupt top-down regulation of arousal and sleep stability, thereby predisposing patients to sleep fragmentation. However, given the cross-sectional nature of the data, these interpretations should be considered cautiously. Conversely, insufficient sleep may further degrade prefrontal functional integrity, establishing a potentially self-reinforcing cycle of dysfunction. This reciprocal relationship highlights the role of the Default_PFCm in both cognitive continuity and sleep regulation.

The co-occurrence of “somatomotor hyper-stability” and “prefrontal default mode instability” points to a coherent pattern of large-scale network dysregulation in the narcoleptic brain. Within the context of normal hierarchical organization, this pattern suggests a hierarchical dissociation, with relatively increased stability in lower-level sensory systems and decreased stability in higher-order cognitive networks. These findings support a model in which NT1 is characterized by a dissociable pattern of network dysfunction across functional levels. Together, these findings suggest a hierarchical imbalance characterized by relative over-stabilization of lower-level systems and reduced temporal integration capacity in higher-order networks. This pattern of dysregulation—reflecting a reduced capacity to flexibly coordinate different neural systems—may represent a potential neural basis for blurred sleep–wake state boundaries. The robustness of this divergent pattern was confirmed through reproducibility analyses, supporting the stability of the observed findings across analytical conditions.

Looking forward, our findings suggest that therapeutic strategies aimed at rebalancing this network stability imbalance—for instance, through interventions targeting large-scale network dynamics (e.g., neuromodulation approaches)—may offer a more nuanced approach than simply promoting global arousal. Certainly, this study has limitations. Its cross-sectional design precludes causal inference; the modest sample size and single-site recruitment limit generalizability. In addition, the supplementary seed-based dFC findings were spatially limited and should be interpreted cautiously. Future studies with larger cohorts should further determine whether these dFC abnormalities preferentially relate to nocturnal sleep fragmentation, daytime sleepiness, or broader clinical phenotypes of NT1. Should the divergent dFC stability track symptom severity in larger cohorts, strategies that rebalance network flexibility—rather than indiscriminately enhancing arousal—may deserve exploration.

In summary, we identify a double dissociation of dynamic functional stability (SMN hyper-rigidity vs. Default_PFCm lability) as a neural signature of NT1. These alterations are further associated with network-specific changes in dynamic functional coupling, linking local stability abnormalities to large-scale network interactions. These opposing alterations offer a parsimonious framework for state instability and may guide future mechanistically grounded interventions.

## Data Availability

The raw data supporting the conclusions of this article will be made available by the authors, without undue reservation.
