# Supplementary material for: Divergent dFC stability of DMN and SMN in narcolepsy
Source: Front Neurosci. 2026 Jun 8;20:1746322. doi: 10.3389/fnins.2026.1746322 (PMC13284077; doi:10.3389/fnins.2026.1746322)
Supplement: Supplementary file 1 [file Supplementary_file_1.docx]

**Supplementary Table 1.** Demographic and clinical characteristics of narcolepsy type 1 (NT1) patients and healthy controls (HCs).

| ID | group | age | gender | Height | Weight | Disease  Duration  （y） | Sleep  Efficiency | SOREMPs | Mean sleep latency | Mean REM latency | MMSE | MoCa | Epworth | ISI | HAMD | HAMA |
| --- | --- | --- | --- | --- | --- | --- | --- | --- | --- | --- | --- | --- | --- | --- | --- | --- |
| NP01 | 1 | 38 | 1 | 176 | 80 | 1 | 63.9 | 4 | 12 | 120 | 29 | 27 | 20 | 3 | 0 | 7 |
| NP02 | 1 | 17 | 2 | 170 | 60 | 6 | 85.1 | 4 | 216 | 420 | 29 | 29 | 19 | 10 | 12 | 15 |
| NP03 | 1 | 38 | 1 | 175 | 75 | 6 | 80.2 | 3 | 302 | 98 | 29 | 22 | 23 | 19 | 9 | 7 |
| NP04 | 1 | 40 | 1 | 170 | 62 | 1 | 73.1 | 5 | 276 | 127 | 30 | 28 | 12 | 16 | 3 | 0 |
| NP05 | 1 | 34 | 1 | 174 | 163 | 10 | 62.1 | 4 | 45 | 106 | 29 | 28 | 22 | 9 | 3 | 0 |
| NP06 | 1 | 15 | 1 | 190 | 115 | 10 | 82.8 | 4 | 203 | 133 | 20 | 12 | 12 | 7 | 7 | 0 |
| NP07 | 1 | 18 | 1 | 187 | 100 | 7 | 52.3 | 2 | 144 | 90 | 29 | 25 | 23 | 16 | 38 | 37 |
| NP08 | 1 | 29 | 1 | 178 | 97 | 13 | 76.3 | 4 | 72 | 114 | 30 | 28 | 19 | 14 | 0 | 0 |
| NP09 | 1 | 25 | 1 | 155 | 110 | 12 | 72.1 | 5 | 72 | 102 | 30 | 29 | 21 | 20 | 1 | 2 |
| NP10 | 1 | 36 | 2 | 175 | 80 | 22 | 68.4 | 3 | 122 | 135 | 29 | 27 | 18 | 5 | 0 | 0 |
| NP11 | 1 | 23 | 1 | 169 | 75 | 12 | 94 | 4 | 108 | 172 | 29 | 24 | 16 | 13 | 1 | 16 |
| NP12 | 1 | 26 | 1 | 183 | 110 | 12 | 70.1 | 4 | 68 | 221 | 28 | 26 | 17 | 8 | 0 | 0 |
| NP13 | 1 | 17 | 1 | 173 | 75 | 7 | 85 | 4 | 636 | 60 | 29 | 29 | 21 | 11 | 1 | 2 |
| NP14 | 1 | 23 | 1 | 173 | 72 | 10 | 88.5 | 5 | 150 | 132 | 28 | 25 | 14 | 11 | 2 | 3 |
| NP15 | 1 | 21 | 1 | 180 | 85 | 2 | 71.9 | 5 | 72 | 252 | 28 | 26 | 6 | 11 | 0 | 0 |
| NP16 | 1 | 50 | 2 | 155 | 63 | 4 | 79 | 4 | 144 | 210 | 28 | 27 | 22 | 13 | 6 | 3 |
| NP17 | 1 | 18 | 2 | 160 | 65 | 4 | 90.7 | 4 | 174 | 56 | 27 | 27 | 12 | 5 | 2 | 1 |
| NP18 | 1 | 11 | 2 | 157 | 56 | 1 | 70.4 | 4 | 60 | 36 | 29 | 27 | 12 | 11 | 0 | 0 |
| NP19 | 1 | 20 | 2 | 159 | 52 | 3 | 71.1 | 2 | 79 | 120 | 28 | 27 | 19 | 6 | 11 | 4 |
| NP20 | 1 | 15 | 2 | 165 | 75 | 2 | 72.1 | 4 | 322 | 392 | 27 | 26 | 13 | 14 | 8 | 6 |
| NP21 | 1 | 36 | 2 | 158 | 65 | 1 | 79.2 | 5 | 414 | 66 | 30 | 29 | 22 | 11 | 5 | 6 |
| NP22 | 1 | 17 | 1 | 170 | 62 | 3 | 80.2 | 4 | 510 | 188 | 29 | 28 | 15 | 7 | 0 | 0 |
| NP23 | 1 | 41 | 2 | 165 | 65 | 2 | 74 | 5 | 126 | 165 | 28 | 25 | 14 | 8 | 18 | 12 |
| NP24 | 1 | 42 | 1 | 175 | 80 | 1 | 78.9 | 2 | 294 | 600 | 28 | 27 | 14 | 16 | 17 | 19 |
| NP25 | 1 | 37 | 1 | 179 | 75 | 9 | 78 | 5 | 168 | 150 | 27 | 26 | 22 | 20 | 12 | 43 |
| NP26 | 1 | 49 | 2 | 171 | 60 | 8 | 81.2 | 5 | 501 | 180 | 28 | 28 | 15 | 19 | 2 | 0 |
| NP27 | 1 | 19 | 1 | 156 | 65 | 8 | 66.3 | 5 | 186 | 246 | 28 | 26 | 15 | 19 | 0 | 1 |
| NC01 | 2 | 38 | 2 |  |  |  |  |  |  |  |  |  |  |  |  |  |
| NC02 | 2 | 37 | 1 |  |  |  |  |  |  |  |  |  |  |  |  |  |
| NC03 | 2 | 36 | 1 |  |  |  |  |  |  |  |  |  |  |  |  |  |
| NC04 | 2 | 33 | 1 |  |  |  |  |  |  |  |  |  |  |  |  |  |
| NC05 | 2 | 22 | 1 |  |  |  |  |  |  |  |  |  |  |  |  |  |
| NC06 | 2 | 25 | 1 |  |  |  |  |  |  |  |  |  |  |  |  |  |
| NC07 | 2 | 26 | 2 |  |  |  |  |  |  |  |  |  |  |  |  |  |
| NC08 | 2 | 21 | 1 |  |  |  |  |  |  |  |  |  |  |  |  |  |
| NC09 | 2 | 23 | 1 |  |  |  |  |  |  |  |  |  |  |  |  |  |
| NC10 | 2 | 24 | 2 |  |  |  |  |  |  |  |  |  |  |  |  |  |
| NC11 | 2 | 21 | 2 |  |  |  |  |  |  |  |  |  |  |  |  |  |
| NC12 | 2 | 21 | 1 |  |  |  |  |  |  |  |  |  |  |  |  |  |
| NC13 | 2 | 20 | 2 |  |  |  |  |  |  |  |  |  |  |  |  |  |
| NC14 | 2 | 21 | 2 |  |  |  |  |  |  |  |  |  |  |  |  |  |
| NC15 | 2 | 22 | 1 |  |  |  |  |  |  |  |  |  |  |  |  |  |
| NC16 | 2 | 20 | 2 |  |  |  |  |  |  |  |  |  |  |  |  |  |
| NC17 | 2 | 21 | 1 |  |  |  |  |  |  |  |  |  |  |  |  |  |
| NC18 | 2 | 22 | 1 |  |  |  |  |  |  |  |  |  |  |  |  |  |
| NC19 | 2 | 22 | 1 |  |  |  |  |  |  |  |  |  |  |  |  |  |
| NC20 | 2 | 19 | 2 |  |  |  |  |  |  |  |  |  |  |  |  |  |
| NC21 | 2 | 27 | 2 |  |  |  |  |  |  |  |  |  |  |  |  |  |
| NC22 | 2 | 16 | 2 |  |  |  |  |  |  |  |  |  |  |  |  |  |
| NC23 | 2 | 16 | 1 |  |  |  |  |  |  |  |  |  |  |  |  |  |
| NC24 | 2 | 14 | 1 |  |  |  |  |  |  |  |  |  |  |  |  |  |
| NC25 | 2 | 32 | 1 |  |  |  |  |  |  |  |  |  |  |  |  |  |

**Abbreviations:** NT1, narcolepsy type 1; HCs, healthy controls; SOREMPs, sleep-onset rapid eye movement periods; MMSE, Mini-Mental State Examination; MoCA, Montreal Cognitive Assessment.

**Notes:** Group code: 1 = NT1 patient, 2 = Healthy control. Gender code: 1 = Male, 2 = Female.
