# Supplementary material for: Divergent dFC stability of DMN and SMN in narcolepsy
Source: Front Neurosci. 2026 Jun 8;20:1746322. doi: 10.3389/fnins.2026.1746322 (PMC13284077; doi:10.3389/fnins.2026.1746322)
Supplement: Supplementary file 2 [file Supplementary_file_2.docx]

**Supplementary Table 2.** Significant clusters showing between-group differences in dFC stability.

| regions | voxels | Yeo2011_7Networks | x | y | z | Peak intensity | Peak  Index |
| --- | --- | --- | --- | --- | --- | --- | --- |
| RH_SomMot | 261 | 17Networks_3 | 6 | -20 | 65 | 5.96 | 6449 |
| RH_Default_PFCm | 41 | 17Networks_16 | 10 | 48 | 13 | -6.03 | 6681 |
| RH_Default_PFCm | 31 | 17Networks_17/16 | 14 | 36 | 48.5 | -4.87 | 8658 |
| LH_SomMot | 329 | 17Networks_3 | -8.5 | -18.5 | 73 | 5.05 | 2109 |
| LH_SomMot/LH_DorsAttn_Post | 46 | 17Networks_3/6 | -34.7 | -37 | 54.5 | 5.5 | 4581 |
| LH_Default_PFC | 40 | 17Networks_17 | -14.7 | 48.5 | 35.4 | -4.77 | 2801 |
| LH_Default_PFC | 24 | 17Networks_16 | -9 | 49 | 8 | -4.84 | 2164 |

**Abbreviations:** dFC, dynamic functional connectivity; MNI, Montreal Neurological Institute; LH, left hemisphere; RH, right hemisphere; SomMot, somatomotor network; Default_PFC(m), (medial) prefrontal default mode network; DorsAttn, dorsal attention network.

**Statistical notes:** Clusters were identified using a voxel-wise threshold of p<0.001p < 0.001p<0.001 and a cluster-level family-wise error (FWE) correction of p<0.05p < 0.05p<0.05 (via Monte Carlo simulation). Peak intensities represent the maximum t-value within the cluster.
