# Supplementary material for: Divergent dFC stability of DMN and SMN in narcolepsy
Source: Front Neurosci. 2026 Jun 8;20:1746322. doi: 10.3389/fnins.2026.1746322 (PMC13284077; doi:10.3389/fnins.2026.1746322)
Supplement: Supplementary file 3 [file Supplementary_file_3.docx]

**Supplementary Table 3**. Cluster-wise results of seed-based dynamic functional connectivity analysis

| **Seed ROI** | **Hemisphere** | **Cluster ID** | **Vertices** | **Area**  **(mm²)** | **Peak MNI**  **(X, Y, Z)** | **Peak Statistic** | **Network Label** |
| --- | --- | --- | --- | --- | --- | --- | --- |
| LH2109 | LH | C1 | 8 | 88.60 | -42.0, -74.3, -2.8 | 4.61 | Visual |
| LH2109 | LH | C2 | 3 | 43.21 | -46.9, -8.3, -31.1 | 3.89 | Default (Temporal) |
| LH2109 | RH | C3 | 4 | 45.62 | 48.0, 37.8, -3.5 | 4.40 | Default / Control |
| RH6449 | RH | C1 | 3 | 45.54 | 44.6, -66.9, -8.6 | 3.92 | Visual |
| RH6681 | RH | C1 | 7 | 49.48 | 33.8, -43.6, 52.0 | 4.70 | Dorsal Attention |

Note: Clusters were identified using vertex-wise analysis with multiple comparison correction (Monte Carlo corrected). Network labels were assigned based on Schaefer 400-parcel atlas and Yeo 17-network parcellation. Only clusters surviving correction are reported.
