# Supplementary material for: Divergent dFC stability of DMN and SMN in narcolepsy
Source: Front Neurosci. 2026 Jun 8;20:1746322. doi: 10.3389/fnins.2026.1746322 (PMC13284077; doi:10.3389/fnins.2026.1746322)
Supplement: Supplementary file 4 [file Supplementary_file_4.docx]

**Supplementary Table 4**. Correlations Between dFC Stability Indices and Clinical Variables in NT1 Patients

| **ROI**  **(Peak Index)** | **Brain Region** | **Clinical Variable** | ***r*** | ***p***  **(uncorrected)** | ***P* _FDR_** |
| --- | --- | --- | --- | --- | --- |
| LH2109 | Left Somatomotor Cortex | Sleep Efficiency | -0.545 | **0.0033** | 0.0116 |
| RH6449 | Right Somatomotor Cortex | Sleep Efficiency | -0.537 | **0.0039** | 0.0116 |
| RH6681 | Right Medial Prefrontal Cortex | Sleep Efficiency | 0.456 | **0.0167** | 0.0292 |
| LH2109 | Left Somatomotor Cortex | ESS | 0.412 | **0.0321** | 0.118 |
| RH6449 | Right Somatomotor Cortex | ESS | 0.398 | **0.0415** | 0.1264 |
| RH6681 | Right Medial Prefrontal Cortex | ESS | -0.361 | 0.0672 | 0.1521 |
| LH2109 | Left Somatomotor Cortex | Mean Sleep Latency | -0.428 | 0.0245 | 0.1093 |
| RH6449 | Right Somatomotor Cortex | Mean Sleep Latency | -0.401 | 0.0384 | 0.1264 |
| RH6681 | Right Medial Prefrontal Cortex | Mean Sleep Latency | 0.336 | 0.0876 | 0.1715 |
| LH2109 | Left Somatomotor Cortex | SOREMPs | 0.287 | 0.1462 | 0.2287 |
| RH6449 | Right Somatomotor Cortex | SOREMPs | 0.301 | 0.1284 | 0.2287 |
| RH6681 | Right Medial Prefrontal Cortex | SOREMPs | -0.255 | 0.1987 | 0.2649 |
| LH2109 | Left Somatomotor Cortex | ISI | 0.388 | 0.0461 | 0.1264 |
| RH6449 | Right Somatomotor Cortex | ISI | 0.371 | 0.0582 | 0.1455 |
| RH6681 | Right Medial Prefrontal Cortex | ISI | -0.344 | 0.0793 | 0.1682 |
| LH2109 | Left Somatomotor Cortex | HAMD | 0.214 | 0.2841 | 0.3312 |
| RH6449 | Right Somatomotor Cortex | HAMD | 0.196 | 0.3276 | 0.3514 |
| RH6681 | Right Medial Prefrontal Cortex | HAMD | -0.173 | 0.3874 | 0.3874 |
| LH2109 | Left Somatomotor Cortex | HAMA | 0.241 | 0.2255 | 0.3079 |
| RH6449 | Right Somatomotor Cortex | HAMA | 0.228 | 0.2536 | 0.319 |
| RH6681 | Right Medial Prefrontal Cortex | HAMA | -0.184 | 0.3598 | 0.3598 |
